# Father Involvement in Pregnancy and Postnatal Care: Combined Perspectives of Fathers, Mothers, and Service Providers

**DOI:** 10.1111/nhs.70105

**Published:** 2025-04-20

**Authors:** Alice Small, Shane A Kavanagh, Jacqui A Macdonald, Laura Di Manno, Karen Wynter

**Affiliations:** ^1^ School of Health & Social Development Faculty of Health, Deakin University Geelong Victoria Australia; ^2^ SEED Lifespan Strategic Research Centre, School of Psychology, Faculty of Health Deakin University Geelong Victoria Australia; ^3^ Department of Paediatrics University of Melbourne Melbourne Victoria Australia; ^4^ Murdoch Children's Research Institute Melbourne Victoria Australia; ^5^ Department of Psychiatry, School of Clinical Sciences Monash University Melbourne Victoria Australia; ^6^ School of Nursing and Midwifery, Faculty of Health Deakin University Geelong Victoria Australia

**Keywords:** fatherhood, health services accessibility, postnatal care, prenatal care

## Abstract

Involving fathers in pregnancy and postnatal healthcare services can enhance family wellbeing, yet father‐inclusive practice remains limited. This study explored the perspectives of key stakeholders (fathers, mothers and service providers) regarding father‐inclusive healthcare. Separate online focus groups were held with Australian fathers (*n* = 4) and mothers (*n* = 10) of infants up to 12 months old. Semi‐structured interviews were held with postnatal service providers (*n* = 12). Each dataset was analyzed thematically; key findings were then synthesized into overarching themes. All participant groups acknowledged the benefits of father involvement. Barriers to father involvement included traditional role divisions, fathers' competing commitments, workplace inflexibilities, and healthcare systems focused on mothers. Enablers included partner support, positive healthcare experiences, and the provision of father‐specific services. Consistent with the view that active fathering has benefits for all the family and to enhance family health, father inclusion is recommended. However, this may require changes at the service, workplace, and societal levels.

**Summary**

- Fathers are seldom actively involved in pregnancy and postnatal healthcare services, despite potential benefits of father involvement for the whole family.
- Mothers, fathers, and service providers recognize there would mostly be benefits from involving fathers in healthcare services, such as fostering shared responsibilities and better support for mothers, and this aligns with modern parenting preferences. There are some circumstances, however, where father involvement may not be recommended.
- To facilitate greater participation of fathers, changes in healthcare services, workplaces, and broader social levels are needed. This includes recognizing the barriers and enablers of father involvement.

## 1 Introduction

The transition to parenthood can be a challenging period for fathers with potential mental health impacts on themselves and their families (Baldwin et al. 2018). Poor mental health in fathers is associated with maternal postpartum depression and poorer mental health, social, emotional, and behavioral development in children later in life (Sweeney and MacBeth 2016). Involving fathers in pregnancy and postnatal healthcare services, therefore, offers an opportunity to assess the health of fathers and improve family health outcomes (Fisher et al. 2021) and may improve the uptake of postnatal care by women (Finlayson et al. 2023).

Internationally, pregnancy and postnatal services have traditionally focused on maternal and infant health. The World Health Organization (WHO), however, calls for male involvement during the perinatal period to support women's access to healthcare and promote men's positive involvement as partners and fathers (WHO 2015). Yet, embedded father‐inclusive practice is often not realized in many healthcare organizational frameworks and services (Hodgson et al. 2021; Høgmo et al. 2021).

Many potential factors affecting father involvement in healthcare services have been identified (Macdonald et al. 2022). Individual and familial factors that can improve father engagement include fathers' positive beliefs and expectations about parenting and help‐seeking (Venning et al. 2021), maternal encouragement (Gervais et al. 2016) and fathers' awareness of available supports (Baldwin et al. 2018). Healthcare service inhibitors include organizational policies, opening hours and limited information or services for fathers (Gervais et al. 2016; Rominov et al. 2017; Wynter et al. 2021), gendered language which is exclusive of partner involvement (Hodgson et al. 2021), poor staff attitudes and limited experience and training in father‐inclusive practice (Wynter et al. 2021). Embedded social structures and traditional “masculine” values may also induce unconscious bias against men as caregivers and contribute to marginalization of men in services (Darwin et al. 2021; Pedersen et al. 2021).

Increasing father involvement is a complex challenge, and gaining a full understanding of the barriers and enablers to father‐inclusive practice requires an understanding of the perspectives of all stakeholders. Previous qualitative studies have explored fathers' perspectives (Baldwin et al. 2018; Finlayson et al. 2023), service providers' perspectives (Buek et al. 2021; Rominov et al. 2017; Wynter et al. 2021), service provider and fathers' perspectives (Pfitzner et al. 2018) and fathers' and mothers' perspectives during pregnancy (Walsh et al. 2021). No recent qualitative studies, however, explore father involvement in both pregnancy and postnatal services to 12 months following birth from the perspectives of fathers, mothers and service providers. Doing so affords an opportunity to inform service delivery in a way that is sensitive to the needs and informed by the insights of all participants in the process. This study, therefore, aimed to investigate perspectives of father involvement in both pregnancy and postnatal healthcare services among fathers, mothers, and service providers to identify barriers and enablers to father‐inclusive healthcare provision. Importantly, we recognize that since families can take many forms, this study's findings are limited to fathers as non‐birthing partners.

## 2 Methods

### 2.1 Study Design

The study used a qualitative approach to investigate perspectives and experiences of three participant groups: fathers (focus group, *n* = 4), mothers (focus groups, *n* = 10), and service providers (interviews, *n* = 12). Data from participants in each group were collected and separately analyzed. The findings were then synthesized by identifying the key findings underpinning participant group data and amalgamating these into overarching themes.

### 2.2 Participants, Recruitment, and Settings

Fathers and mothers were recruited via convenience sampling from Maternal and Child Health (MCH) services in Victoria, Australia. These services are offered free of charge to all Victorian families with children, from birth until 4 to 5 years old. A research flyer was included in the state‐wide MCH newsletter and shared in social media networks of the research team, inviting parents to participate in focus groups. Eligible participants were fathers and mothers with an infant 12 months or younger. Consent to participate was given by participants in an Expression of Interest Qualtrics survey with an attached Plain Language Statement (PLS). An anonymous, non‐compulsory demographic survey was conducted at the beginning of each focus group using the Polls function in Zoom (see Table 1).

**TABLE 1:** Demographic characteristics of fathers and mothers.

	Fathers (*n* = 4)	Mothers (*n* = 10)
Age range (years)	31–39	30–40
First‐time parents	4	7
Age range of infant (in months)	1.5–8	2–8

Service providers were recruited via purposive sampling from two Victorian healthcare programs known to explicitly include fathers in their services. Some of these participants were from a couple‐focused program promoting healthy and respectful relationships among new parents. This program was incorporated in First‐Time Parent Groups presented at some MCH services centers. Other participants were recruited from a residential early parenting service helping families with children up to 4 years, commonly with sleep and settling difficulties. Participants were emailed a PLS and consent form, which were completed and returned to the research team before interviews.

### 2.3 Data Collection

Interview and focus group discussion guides were developed by a team of fatherhood research experts, informed by the research aims and relevant literature. Data were collected during the period 2022–2023 from fathers and mothers via focus groups conducted online using Zoom, which afforded convenience for participants and geographic location coverage (Stewart and Shamdasani 2016). Groups for fathers and mothers were run separately to cultivate meaningful group discussion and cohesion among participants groups. Focus groups ran for 60 min and included four participants in one fathers' focus group, and five participants in each of the two mothers' groups. The initial aim was to conduct two focus groups for both mother and father participants, but owing to difficulties recruiting fathers, only one father focus group was conducted. Automated zoom transcripts for each focus group were then downloaded, checked for accuracy, and corrected as appropriate by the first author.

Data from service providers were collected during the period 2018–2019 via semi‐structured interviews as clinical commitments made participation in a focus group during workdays unlikely. Thirteen semi‐structured interviews were conducted, either face‐to‐face or by telephone, with a duration of 20–60 min. Interviews were audio recorded and transcribed verbatim by the research team. One participant's interview was subsequently excluded from the data analysis as the audio recording was inaudible.

Originally, data collection was planned for 2018–2019, and fathers were to be recruited from the same two organizations as service providers. A restructuring occurred at both service organizations at the end of 2018, making recruitment of fathers via these organizations challenging. This necessitated a change to the planned recruitment, which was further impacted by the COVID‐19 pandemic. Thus, study findings were drawn from data collected from service providers in 2018–2019 and from fathers and mothers via universal MCH services in 2022–2023.

### 2.4 Data Analysis

Participant group data were analyzed separately using inductive enquiry, and patterns of meaning were identified using Reflexive Thematic Analysis (RTA) (Braun and Clarke 2022). RTA allows flexibility in analyzing the different participant group sizes and data collection methods (Braun and Clarke 2020). A critical realist stance was taken to allow for pragmatic exploration of data and to gain further insights into the influence of the many contextual factors that are relevant to participants (Pilgrim 2014).

Synthesis of the key findings from participant group data was then conducted to form comprehensive themes which provide a picture of participants' experiences and an overarching combination of the key findings with respect to the research aims (O'Reilly et al. 2021). As the researcher's own perspectives on the interpretation of data need to be considered when reflecting on the understandings that have been developed (Mitchell et al. 2018), we declare that the first author identifies as a female who has recent lived experience utilizing maternity services in Victoria, Australia. Collaboration with the research team and a reflexive journal were maintained throughout the process of data collection and analysis to allow reflection on assumptions, expectations, and choices (Braun and Clarke 2022).

## 3 Results

Demographic characteristics of the parents and service providers are presented in Tables 1 and 2, respectively.

**TABLE 2:** Demographic and professional characteristics of service providers.

	Frequency (*n* = 12)
Gender	
Female	10
Male	2
Profession	
Nursing	3
Group facilitator	3
Manager	4
Medical	2
Years in profession	
1–5 years	2
6–10 years	5
11–20 years	3
21–30 years	2

**FIGURE 1:**
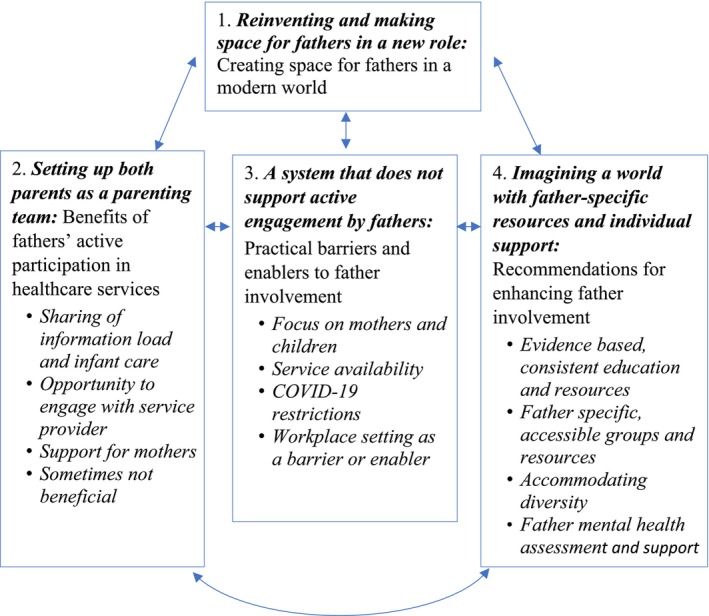
Themes from father, mother, and service provider data synthesis.

Four themes were identified from all three participant groups' data synthesis as follows: reinventing and making space for fathers in a new role; setting up both parents as a parenting team; a system that does not support active engagement by fathers; and imagining a world with father‐specific resources and individual support (Figure 1).

### 3.1 Theme 1: Reinventing, and Making Space for, Fathers in a New Role

An overarching finding across all participant groups concerned the shifting of cultural norms around fathers' roles.

Service providers noted an increasing awareness of the important influence fathers have on the wellbeing of mothers and children and the need for greater father‐inclusion in services:

If we want to support women to be able to have the choices to, you know, around their health, around their well‐being and their lives we actually have to make sure that the men are identified and supported and helped too [SP12].

This was consistent with fathers' views of themselves as engaged in parenting and sharing the division of care tasks with their partners; they recognized that this differs from the traditional, predominant view and from gendered parenting norms of “mothers as carers, fathers as workers”:

So, I might not be the average father, maybe none of us are the average father, I'm not sure, but a lot of our friends do a similar thing. The father [is] quite involved and, you know, I think that's something to celebrate [F3].

Similarly, mothers valued the sharing of parenting loads and their “engaged” partners, while acknowledging that this may not be the norm as stigmas exist around fathers being involved in caregiving:

… there'd probably be a little bit of stigma to say… ‘I may be a stay‐at‐home dad for a few months’ or ‘I'm going to take some time off work to support my partner with a new child’. I think there'd be a little bit of that which is really disappointing but unfortunately we haven't developed probably as well as we should yet as a nation [M4].

### 3.2 Theme 2: Setting Up Both Parents as a Parenting Team

Among father and mothers there was a view of active fathering providing the basis for an effective parenting team. Service providers endorsed a family approach in care provision by recognizing and promoting the needs of the family:

… you know it's in the title, ‘family’ centre even though most of our admitted clients are women, you know, those women are, mostly, part of a family unit so the emphasis is on that family unit [SP 6].

Benefits of fathers' participation included information load sharing and infant care, the opportunity to ask questions and discuss strategies with service providers and support for mothers. Most mothers and fathers agreed that attendance at pregnancy and postnatal appointments enhanced “team parenting” by offering a space where fathers could be included in the care of their infant:

Thought it was very useful to get across the same information that she got because we both wanted to be the same level of informed [F4].

It sets up an expectation really early on that, like the parenting belongs to both parents [M9].

I think just the general feeling of involvement I've noticed when my partner's been able to join for things; he understands more, he feels like he can be more active in this baby … My partner does everything in the appointments, I don't touch my baby when I'm in there because it's his way of getting involved [M5].

Some mothers reported that if fathers were not present at postnatal appointments, this made it difficult to relay and implement advice and strategies from service providers since fathers did not have the opportunity to participate in the discussion with service providers:

When, you know, you come home and try and institute some of the advice that you've been given in these appointments, and it's like, well, it doesn't work for our family, they get it, certainly my husband has a negative view of the maternal, a lot of the maternal nurses, and I think that further disengages them [M8].

Furthermore, when the health of the mother or infant was a concern, partner support became more crucial to mothers:

I suppose just to explain to them [partners] just how afraid they [service providers] make you, rightly so, because they want to protect your baby but he kind of miss[ed] that not being there and not seeing that … I think that if everything is going okay, it's fine, but if something is going wrong, we do need someone [M1].

However, in some circumstances, having fathers attend appointments was not regarded by participants as beneficial. Service providers and mothers cautioned against all partners attending services. For example, for women experiencing intimate partner violence, the healthcare setting can function as a refuge for accessing assistance. One mother indicated:

In that scenario, because of COVID, that the husband not being there for that lady to be able to be completely transparent about what was going on in her life, was actually a very good thing because it was the first time she was actually able to talk about it [M4].

### 3.3 Theme 3: A System That Does Not Support Active Engagement by Fathers

Many practical barriers and enablers to father participation in healthcare services were identified at all focus groups and some factors can both represent barriers and enablers. Factors discussed included the general focus on mothers and children during the perinatal period, restricted service hours, COVID‐19 restrictions and workplace settings.

In general, all participant groups agreed that, for many reasons, health services are predominantly oriented toward the care of women and children. They included the use of exclusive language in services' descriptions (“Maternal and child health” services) and embedded administrative processes (e.g., references to “primary” carer vs. “secondary” carer). This father noted:

With the maternal health nurse, you do, sort of, feel like a “second class citizen” sometimes. I think that [the] focus, definitely, is a lot more on mum and bub, which, again I think, does make perfect sense [F1].

Since healthcare service appointments occurred mainly during business hours, issues of practicality, convenience and time commitments sometimes weighed against partner attendance. Service providers and mothers reported:

We don't see them here at the program because they are doing everything they can to support their wife and family. In the best that they know how. Life I think gets in the way [SP7].

It made sense for me to be going along to appointments and if he was able to get a bit of time off and work from home, and perhaps be able to sneak out with me to a MCH appointment, then he would, but it was more things that I could just take care of [M3].

COVID‐19 restrictions which prevented partners from attending antenatal appointments with their partners, led to mother participants describing their partner as feeling frustrated and disappointed and mothers feeling alone and isolated. While the use of online technology ameliorated this somewhat, mothers felt that face‐to‐face consultations for both parents were vital for a complete experience and engagement in the process:

Oh, it was hard. It was hard for me especially being my first pregnancy, and I had a few complications, to be there on my own [M2].

Fathers who had experienced COVID‐19 restrictions and were unable to attend appointments felt a sense of disconnection from not having a shared experience with partners, while recognizing restrictions were necessary for the health and safety of hospital systems and staff:

I found [there] was quite a big disconnect between my experience of our pregnancy and my wife's experiencing and we tried hard to try and reconnect that [F2].

Some fathers reported that healthcare services accommodated their needs despite COVID‐19 restrictions, for instance, where remote access options were offered, although this differed among participants:

I just also wanted to express surprise that the council from [another father participant] didn't offer any kind of remote Zoom based MCH appointments because I feel like, in mine, that was for a while the case. So different councils, different rules, but I think generally my experience has been really good [F4].

Overall, there was general agreement among mothers that the lack of adequate antenatal education for fathers during the COVID‐19 pandemic left them less prepared for the birth and postnatal periods:

I think the lack of [antenatal education] sessions were a bit frustrating … we watched the pre‐recorded videos together, but it didn't really tell you much. They were good, but it was more like “here's how birth happens” not “here's how you can help through birth” [M5].

Fathers acknowledged that workplace contexts/policies can influence their motivation and ability to participate in caring roles and services. This could be both a barrier and an enabler to father participation in healthcare services. Fathers reported that their workplace context was an enabler, including the opportunity to work from home, a supportive and flexible workplace, and proximity to services. However, for other fathers, workplace arrangements represent a significant barrier to participation:

My brother‐in‐law is a tradie [tradesperson], … he got two weeks off for each of my nieces and he works six days a week and didn't go to any [appointments] but that's the job that he's got, and in that industry, from what he told me, you can't just ask your boss for a few hours off to support [your] wife to go to any of these appointments [F3].

### 3.4 Theme 4: Imagining a World With Father‐Specific Resources and Individual Support

One important enabler related to building in father‐focused components. Suggestions for improving engagement of fathers included the development of evidence‐based education and resources, including father‐specific groups and information accessible in flexible modalities (e.g., online), tailored approaches to meet each family's needs, and awareness of mental health needs among fathers.

Service providers considered father‐inclusion as not lessening the focus on mothers and infants, and supported investing time and effort in facilitating opportunities for fathers to have “space to participate” [SP3] in whatever capacities they could. They felt that father‐inclusion could be strengthened in most care models, for example, by considering acceptable approaches for men in their recruitment into programs:

Having some father‐specific stuff is important. There's quite a lot of research around the different roles that dads and mums play in a child's life being sort of cognisant of that and using some of that research to design programs that are relevant and meaningful [SP6].

Service providers also identified important collaborative support alternatives, such as online and face‐to‐face contact, to meet the diverse needs of fathers and more comprehensively ensure that their perspectives are considered:

The capacity for early parenting centres, Maternal and Child Health services and so on and so on, to work collaboratively to develop a suite of services for fathers that can essentially be used across multiple locations, organisation, groups, and so on [SP1].

Mothers considered First Time Parent Groups a worthy initiative for supporting mothers and, for some, their partners as well. They recommended that specific and separate support networks for fathers may also be a viable option for encouraging men to be involved:

A networking session for fathers or even down at the local pub. I don't know, I suppose, like a father's group, perhaps, and they would just if they wanted to rock up just yeah, they're [a] closed book, really a lot of them [M6].

Fathers also indicated that antenatal education should include more father‐specific advice relating to the birth and postnatal periods, to better prepare them for the experience of birth, and role divisions and to increase their knowledge and coping capacity. As this father highlighted, the experience of the transition to fatherhood can be quite stressful:

[We] kind of talked more about what our roles were and I think that's from speaking with other friends who are parents of young kids, they really struggled with how, what do I, [and] what should I be doing [because] all I want to do is the right thing? How can I help my wife and how can I help my child [and] so better preparation around that, I think, would be helpful for dads [F2].

Service providers observed that considering and accommodating diverse family values, cultural and parenting preferences is likely to influence participation and engagement:

It's not one size fits all [SP4].

Fathers stressed the need for their own mental health support, expressing surprise that this was regarded as less important than support for their partners:

There's a big focus, which is great, on mental health in mothers. I think when it was broached with me it was an afterthought, not that I think we've [not] both been cop[ing] with it quite well, but I was really surprised that there's so much focus on the mother's mental health that's not extended to the father because, obviously, that can create problems in the home itself, so I was quite surprised [F2].

## 4 Discussion

The endorsement of father inclusion across all participant groups is a hopeful sign that it is possible to build services that are jointly valued by fathers, mothers, and service providers. Service providers considered father involvement important for family health and well‐being, and although working in postnatal settings, they considered it valuable to maximize collaborative father‐inclusive practice across antenatal and postnatal services. Mothers highly valued partner support, particularly when there were health concerns. Both parent groups identified that involving fathers in healthcare services can facilitate fathers' bonding with their infant and confidence in sharing care tasks. This aligns with research demonstrating that engaging men in health services can improve couple communication and decision‐making (Finlayson et al. 2023) and also suggests that father‐inclusive practice resonates with a new emerging view of engaged parenting during fatherhood. This study, for the first time, included mothers' insights on father involvement in postnatal services and therefore adds to existing research regarding mothers' preferences. Consistent with research on mothers' perspectives during pregnancy (Walsh et al. 2021), mothers endorsed father participation as both an important act of partner support and an act of assuming the role of father.

Yet, the results suggest that substantial change is required at the service level and in culture more broadly, with a complex array of interrelated factors highlighted that function as barriers and enablers for father involvement in services. Familial preferences and how parents choose to parent, for example, can both facilitate and impede father involvement. Consistent with existing evidence (Rominov et al. 2017; Wynter et al. 2021), service providers appeared to understand that the context of family structures would impact their service delivery, requiring them to adapt accordingly to meet the needs of each family. Factors that enhanced engagement included service availability, proximity to services, and consistency in education/advice. Barriers included lack of consistency in advice (Pfitzner et al. 2018; Rominov et al. 2018) and limited service hours (Jeffery et al. 2015; Pfitzner et al. 2018; Wells 2016; Wynter et al. 2015). Even though fathers are more likely to be engaged if health professionals treat them as if they have a valued role in decision making (Jeffery et al. 2015; Wells 2016), all participant groups commented on the continued orientation of services around mothers. Similar to existing evidence among fathers (Finlayson et al. 2023; Walsh et al. 2021), each group believed this was an understandable orientation of service delivery but suggested that increased support for fathers would assist both with their health and preference for sharing care tasks among parents.

Mother and father groups' experiences of online inclusion, particularly during COVID‐19 restrictions, were positive. It is possible that offering the choice of remote attendance to partners, both discretionary and for existential workplace and other commitment clashes, is worthy of further exploration. External social and workplace factors (e.g., workplace flexibility and leave entitlements) were identified as crucial to father involvement in services by all participant groups. Fathers in this study were fortunate to have had access to leave entitlements in excess of the national standard of 2 weeks following the birth of their infants. Additionally, some fathers had the ability to work from home, which was seen as enhancing parental participation opportunities. Owing to pervasive Australian work cultural norms, flexible working arrangements which might allow fathers to attend healthcare appointments with their partners are often not accessed by fathers in Australia (Borgkvist et al. 2018). Changes in workplace environments may encourage father involvement (Finlayson et al. 2023; Hodgson et al. 2021), and how father uptake of entitlements translates into father involvement in services merits further research.

Service providers advocated that creating father‐friendly environments, both physically and operationally, is important for facilitating father‐inclusion. Fathers recommended that education could be provided during pregnancy on managing changes which occur in partner relationships once an infant is born. Some men, however, do not feel like fathers until after birth (Baldwin et al. 2018), making antenatal information feels less relevant. Recognizing the varying experiences of men transitioning to fatherhood is therefore needed to tailor antenatal programs aligned with fathers' needs. Evidence also highlights that targeting couples' relationships can reduce parenting stress and improve parenting quality (Feinberg et al. 2016; Xiao and Loke 2021). Consistent with existing research suggesting that fathers would prefer more “hands‐on” advice, with tailored information to them (Baldwin et al. 2018), all participant groups suggested targeted information and resources on father‐specific tasks and responsibilities, as well as strategies to support their partners and infants. Further, formal postnatal father support groups were suggested by mothers as an approach for aiding father involvement. In Victoria, First Time Parent Groups (FTPG) are mostly attended by mothers and colloquially still known as “mothers' groups”; research is needed to identify whether FTPG meet the needs of men and women and other support persons.

One important aspect of father‐inclusion identified by this study and others (Darwin et al. 2021; Fisher et al. 2021; Macdonald et al. 2021; Schuppan et al. 2019) is the need for awareness and assessment of fathers' mental health. All participant groups recognized the need to promote screening and management of paternal mental health. Fathers identified that, compared with their partners, their mental health was not as well acknowledged.

Finally, the results highlight that father‐inclusive approaches need to be considered with caution. While all mother and father study participants were highly engaged in parenting and supporting their partners, one participant noted that a father's presence was not always valued, for example where women were experiencing intimate partner violence. At these times, the healthcare system can function as a significant point of care in women's and children's health and wellbeing (Gartland et al. 2022). There may therefore be circumstances when engaging the father may be inappropriate, implying more complex and problematic individual assessments are needed in each family situation (Lechowicz et al. 2019).

This study has both strengths and limitations. It is the first study to concurrently examine the perspectives of fathers, mothers, and service providers on active father‐inclusion within antenatal and postnatal healthcare services. As well, this is the first study to include mothers' perspectives of father involvement in postnatal services.

Limitations include the sampling of service providers within postnatal services already oriented to father inclusion; this increases bias toward those with positive views of including fathers. Convenience sampling of parents may have introduced similar bias; for example, parent participants were all highly engaged in parenting practices or healthcare services, and all endorsed division of parental responsibilities. In addition, the sample size for fathers was small, reflecting perhaps the imbalance that exists between father and mother engagement in services, and all fathers were first‐time fathers and had taken six or more weeks of employment leave, which is well in excess of the current Government paid parental leave scheme for fathers in Australia. A further limitation relates to data collection from service providers who did not, and from mothers and fathers who did, experience the impacts of the COVID‐19 pandemic, during and after which healthcare systems and service delivery underwent significant changes.

Results should therefore be interpreted with caution as the smaller sample size of fathers, lack of diverse participants, and time difference between datasets together raise questions about the transferability of results (Smith 2018). Larger studies are needed that include service providers in antenatal settings and a more comprehensive range of fathers (such as non‐resident fathers, stay‐at‐home fathers, parents of different ethnic groups), plus same‐sex partners and disparate groups of non‐birthing partners to cover the great diversity that exists in family structures.

## 5 Conclusion

This study included participants highly engaged in parenting and service providers working in father‐inclusive services and, as such, findings need to be considered with caution. Fathers, mothers, and service providers generally supported fathers attending services. Each group provided nuanced perspectives on complex familial factors influencing parents' decisions and the perceived lack of father support in healthcare services and workplaces. Further exploration of options to engage fathers, such as via online options, targeted antenatal father education, and support for fathers' mental health needs, is suggested to help address participation barriers. Investigating workplace factors and paid workplace leave entitlements aligned with family‐inclusive practice could be beneficial to family health outcomes.

After the birth of a child, father involvement may be desired both by fathers and mothers, both for support and as an important facilitator of co‐parenting. While during pregnancy, inclusion of fathers may occur naturally around special events, such as an ultrasound (Walsh et al. 2021), in the postnatal period, mothers, service providers, and fathers agreed that specific father‐focused groups and resources may be necessary to continue father engagement. Although service providers face barriers in providing such options, there is a shared vision for father‐inclusive and/or ‐focused care, not at the expense of, but rather to enhance optimal care for mothers and infants.

### 5.1 Relevance for Clinical Practice

This study supports the importance of father inclusion in health services during pregnancy and postnatal care and adds mothers' preferences for greater father engagement. The findings could help inform policies aimed at promoting family‐centered care, including the creation of father‐friendly environments, tailored education, and addressing fathers' mental health needs. Flexible service delivery options, such as online platforms to address workplace barriers, may further enhance father involvement. These adjustments in healthcare services' practice could strengthen co‐parenting, foster more positive relationships, and ultimately improve family wellbeing.

## Author Contributions

**Alice Small:** writing – original draft, methodology, formal analysis, data curation, conceptualization, investigation, visualization, writing – review and editing, project administration. **Shane A Kavanagh:** writing – review and editing, formal analysis, supervision, methodology, writing – original draft, conceptualization, visualization, investigation. **Jacqui A Macdonald:** conceptualization, funding acquisition, investigation, writing – review and editing, data curation, methodology, supervision. **Laura Di Manno:** funding acquisition, conceptualization, investigation, writing – review and editing, data curation, methodology, project administration. **Karen Wynter:** conceptualization, investigation, funding acquisition, writing – original draft, writing – review and editing, methodology, formal analysis, supervision, visualization, data curation.

## Ethics Statement

This study received low risk ethics approval from the Deakin Human Ethics Advisory Group (HEAG‐H 169_2018).

## Conflicts of Interest

The authors declare no conflicts of interest.

## Data Availability

The data that support the findings of this study are available on request from the corresponding author. The data are not publicly available due to privacy or ethical restrictions.
